# Supplementary material for: Efficacy of probiotics in patients with cognitive impairment: A systematic review and meta-analysis
Source: PLoS One. 2025 May 2;20(5):e0321567. doi: 10.1371/journal.pone.0321567 (PMC12047807; doi:10.1371/journal.pone.0321567)
Supplement: S1 Table — (DOCX) [file pone.0321567.s001.docx]

| **Section and Topic** | **Item #** | **Checklist item** | **Location where item is reported** |
| --- | --- | --- | --- |
| **TITLE** | | |  |
| Title | 1 | Efficacy of probiotics in patients with cognitive impairment: A systematic review and meta-analysis | Page: 1 |
| **ABSTRACT** | | |  |
| Abstract | 2 | **Objective** To conduct an in-depth exploration of the specific impacts of probiotics and prebiotic supplements on cognitive impairment, it is imperative to also investigate pertinent factors, including the optimal dosage of probiotics for enhancing cognitive function. Such an investigation is crucial for the purpose of developing a tailored probiotic intervention program in clinical settings, aimed at preventing and aiding in the treatment of cognitive decline among patients with cognitive impairment. **Methods** A comprehensive computerized search was conducted across the Embase, PubMed, Web of Science, Cochrane Library, SinoMed, CNKI, Wanfang and WeiPu Data, targeting randomized controlled trials (RCTs) that evaluated the efficacy of probiotics in providing cognitive relief. This search covered a timeframe extending from the inception of each database to September 2024. Following an independent process of literature screening, data extraction, and rigorous quality assessment conducted by two investigators, a meta-analysis was performed using Stata 15.0 software. **Results** A total of ten studies, involving 778 patients, were included in the analysis. The meta-analysis revealed that probiotics were effective in enhancing cognitive function among patients with cognitive impairment, with a standardized mean difference (SMD) of 0.52 (95% CI: 0.07, 0.98; P < 0.001). Subgroup analysis further demonstrated that the largest effect size was observed for studies utilizing the Mini-Mental State Examination (MMSE) scale as the outcome measure (SMD = 0.88). Additionally, the greatest efficacy was associated with single-strain probiotics (SMD = 0.81), and interventions lasting ≤12 weeks exhibited the most pronounced effect (SMD = 0.61). **Conclusion** Probiotics have been shown to enhance cognitive function, with a probiotic intervention program featuring a single probiotic strain and a duration of ≤12 weeks demonstrating particularly robust efficacy in improving cognitive function, as assessed by the Mini-Mental State Examination (MMSE) scale. | Pages: 1-2 |
| **INTRODUCTION** | | |  |
| Rationale | 3 | Probiotics are thought to be beneficial for brain health and cognitive function. However, as the evidence is currently inconsistent, some studies have shown improvement in cognitive function and some have shown no benefit to cognitive function. Therefore, further research is needed to clarify the efficacy of probiotics in patients with cognitive impairment. | Pages: 2-5 |
| Objectives | 4 | To conduct an in-depth exploration of the specific impacts of probiotics and prebiotic supplements on cognitive impairment, it is imperative to also investigate pertinent factors, including the optimal dosage of probiotics for enhancing cognitive function. Such an investigation is crucial for the purpose of developing a tailored probiotic intervention program in clinical settings, aimed at preventing and aiding in the treatment of cognitive decline among patients with cognitive impairment. | Pages: 5-6 |
| **METHODS** | | |  |
| Eligibility criteria | 5 | The inclusion criteria for this systematic review were established according to the PICOS framework (Population, Intervention, Comparison, Outcomes, Study design), as detailed in Table 1. Specifically, the inclusion criteria encompassed:①Study Design: Randomized controlled trials (RCTs) were considered;②Population: Participants had to exhibit cognitive impairment;③Intervention: The intervention group received probiotics, whereas the control group underwent a placebo intervention;④Outcome Measures: Primary focus was on cognitive function.The exclusion criteria were as follows:①Duplicate Literature: Articles that were identical to others in the review were excluded;②Language Restriction: Only Chinese and English language publications were included;③Data Accessibility and Quality: Studies that were unavailable in full text, had ambiguous, incomplete, or unconvertible data, or those that could not be merged with other studies were excluded;④Quality Assessment: Studies rated as grade C in the quality evaluation were not included. | Pages: 6-7 |
| Information sources | 6 | We searched PubMed, Embase, Web of Science, the Cochrane Library, CNKI, WeiPu Database, Wanfang Database, and SinoMed. The last search was conducted on September 2024. | Page: 7 |
| Search strategy | 7 | The search strategy employed a combination of subject headings (Mesh terms) and free-text terms.  **(1)Web of Science Search Strategy**  # Web of Science Search Strategy (v0.1)  # Database: All Databases  # Entitlements:  - WOS: 1985 to 2024  - BIOSIS: 2011 to 2024  - CSCD: 1989 to 2024  - KJD: 1980 to 2024  - MEDLINE: 1950 to 2024  - PPRN: 1991 to 2024  - PQDT: 1637 to 2024  - SCIELO: 2002 to 2024  # Searches:  1: Cognitive Dysfunction (Topic) OR Cognitive Dysfunctions (Topic) OR Dysfunction, Cognitive  (Topic) OR Dysfunctions, Cognitive (Topic) OR Cognitive Impairments (Topic) OR Cognitive  Impairment (Topic) OR Impairment, Cognitive (Topic) OR Impairments, Cognitive (Topic) OR  Cognitive Disorder (Topic) OR Cognitive Disorders (Topic) OR Disorder, Cognitive (Topic) OR  Disorders, Cognitive (Topic) OR Mild Cognitive Impairment (Topic) OR Cognitive Impairment,  Mild (Topic) OR Cognitive Impairments, Mild (Topic) OR Impairment, Mild Cognitive (Topic) OR Impairments, Mild Cognitive (Topic) OR Mild Cognitive Impairments (Topic) OR Cognitive  Decline (Topic) OR Cognitive Declines (Topic) OR Decline, Cognitive (Topic) OR Declines,  Cognitive (Topic) OR Mental Deterioration (Topic) OR Deterioration, Mental (Topic) OR  Deteriorations, Mental (Topic) OR Mental Deteriorations (Topic) and Preprint Citation Index  (Exclude – Database) Date Run: Fri Sep 09 2024 14:25:23 GMT+0800  (GMT+08:00) Results: 526454  2: Probiotics (Topic) OR Probiotic (Topic) and Preprint Citation Index (Exclude –  Database) Date Run: Fri Sep 09 2024 14:26:26 GMT+0800 (GMT+08:00) Results: 82020  3: ((((TS=(randomized controlled trial)) OR TS=(randomized)) OR TS=(placebo)) OR  TS=(randomised)) OR TS=(random) and Preprint Citation Index (Exclude – Database) Date  Run: Fri Sep 09 2024 14:32:44 GMT+0800 (GMT+08:00) Results: 2742637  4: #1 AND #2 AND #3 and Preprint Citation Index (Exclude – Database) Date Run: Fri Sep  09 2024 14:34:51 GMT+0800 (GMT+08:00) Results: 222  **(2)Embase**  Session Results  .......................................................  No. Query Results Results Date  #8. #3 AND #6 AND #7 99 Sep 2024  #7. 'randomized controlled trial':ab,ti OR 1,159,505 Sep 2024  'randomized':ab,ti OR 'placebo':ab,ti  #6. #4 OR #5 63,736 Sep 2024  #5. 'probiotic':ab,ti OR 'probiotics':ab,ti 47,356 Sep 2024  #4. 'probiotic agent'/exp 54,599 Sep 2024  #3. #1 OR #2 660,378 Sep 2024  #2. 'cognitive dysfunctions':ab,ti OR 'dysfunction, 183,972 Sep 2024  cognitive':ab,ti OR 'dysfunctions,  cognitive':ab,ti OR 'cognitive impairments':ab,ti  OR 'cognitive impairment':ab,ti OR 'impairment,  cognitive':ab,ti OR 'impairments,  cognitive':ab,ti OR 'cognitive disorder':ab,ti OR  'cognitive disorders':ab,ti OR 'disorder,  cognitive':ab,ti OR 'disorders, cognitive':ab,ti  OR 'mild cognitive impairment':ab,ti OR  'cognitive impairment, mild':ab,ti OR 'cognitive  impairments, mild':ab,ti OR 'impairment, mild  cognitive':ab,ti OR 'impairments, mild  cognitive':ab,ti OR 'mild cognitive  impairments':ab,ti OR 'cognitive decline':ab,ti  OR 'cognitive declines':ab,ti OR 'decline,  cognitive':ab,ti OR 'declines, cognitive':ab,ti  OR 'mental deterioration':ab,ti OR  'deterioration, mental':ab,ti OR 'deteriorations,  mental':ab,ti OR 'mental deteriorations':ab,ti  #1. 'cognitive defect'/exp 628,449 Sep 2024  **(3)Cochrane Library**  Search Name: 检索历史  Date Run: 07/09/2024 22:16:52  Comment:  ID Search Hits  #1 MeSH descriptor: [Cognitive Dysfunction] explode all trees 3159  #2 (Cognitive Dysfunctions):ti,ab,kw OR (Dysfunction, Cognitive):ti,ab,kw OR (Dysfunctions, Cognitive):ti,ab,kw OR (Cognitive Impairments):ti,ab,kw OR (Cognitive Impairment):ti,ab,kw OR (Impairment, Cognitive):ti,ab,kw OR (Impairments, Cognitive):ti,ab,kw OR (Cognitive Disorder):ti,ab,kw OR (Cognitive Disorders):ti,ab,kw OR (Disorder, Cognitive):ti,ab,kw OR (Disorders, Cognitive):ti,ab,kw OR (Mild Cognitive Impairment):ti,ab,kw OR (Cognitive Impairment, Mild):ti,ab,kw OR (Cognitive Impairments, Mild):ti,ab,kw OR (Impairment, Mild Cognitive):ti,ab,kw OR (Impairments, Mild Cognitive):ti,ab,kw OR (Mild Cognitive Impairments):ti,ab,kw OR (Cognitive Decline):ti,ab,kw OR (Cognitive Declines):ti,ab,kw OR (Decline, Cognitive):ti,ab,kw OR (Declines, Cognitive):ti,ab,kw OR (Mental Deterioration):ti,ab,kw OR (Deterioration, Mental):ti,ab,kw OR (Deteriorations, Mental):ti,ab,kw OR (Mental Deteriorations):ti,ab,kw 53629  #3 #1 OR #2 53640  #4 MeSH descriptor: [Probiotics] explode all trees 3122  #5 (Probiotic):ti,ab,kw 7159  #6 #4 OR #5 8040  #7 #3 AND #6 123  **(4)Pubmed**  #7 (("Probiotics"[Mesh]) OR (Probiotic[Title/Abstract])) AND (("Cognitive Dysfunction"[Mesh]) OR (((((((((((((((((((((((((Cognitive Dysfunctions[Title/Abstract]) OR (Dysfunction, Cognitive[Title/Abstract])) OR (Dysfunctions, Cognitive[Title/Abstract])) OR (Cognitive Impairments[Title/Abstract])) OR (Cognitive Impairment[Title/Abstract])) OR (Impairment, Cognitive[Title/Abstract])) OR (Impairments, Cognitive[Title/Abstract])) OR (Cognitive Disorder[Title/Abstract])) OR (Cognitive Disorders[Title/Abstract])) OR (Disorder, Cognitive[Title/Abstract])) OR (Disorders, Cognitive[Title/Abstract])) OR (Mild Cognitive Impairment[Title/Abstract])) OR (Cognitive Impairment, Mild[Title/Abstract])) OR (Cognitive Impairments, Mild[Title/Abstract])) OR (Impairment, Mild Cognitive[Title/Abstract])) OR (Impairments, Mild Cognitive[Title/Abstract])) OR (Mild Cognitive Impairments[Title/Abstract])) OR (Cognitive Decline[Title/Abstract])) OR (Cognitive Declines[Title/Abstract])) OR (Decline, Cognitive[Title/Abstract])) OR (Declines, Cognitive[Title/Abstract])) OR (Mental Deterioration[Title/Abstract])) OR (Deterioration, Mental[Title/Abstract])) OR (Deteriorations, Mental[Title/Abstract])) OR (Mental Deteriorations[Title/Abstract]))) ("Probiotics"[MeSH Terms] OR "Probiotic"[Title/Abstract]) AND ("Cognitive Dysfunction"[MeSH Terms] OR ("cognitive dysfunctions"[Title/Abstract] OR "dysfunction cognitive"[Title/Abstract] OR "dysfunctions cognitive"[Title/Abstract] OR "cognitive impairments"[Title/Abstract] OR "cognitive impairment"[Title/Abstract] OR "impairment cognitive"[Title/Abstract] OR "impairments cognitive"[Title/Abstract] OR "cognitive disorder"[Title/Abstract] OR "cognitive disorders"[Title/Abstract] OR "disorder cognitive"[Title/Abstract] OR "disorders cognitive"[Title/Abstract] OR "mild cognitive impairment"[Title/Abstract] OR "cognitive impairment mild"[Title/Abstract] OR "cognitive impairments mild"[Title/Abstract] OR "impairment mild cognitive"[Title/Abstract] OR "impairments mild cognitive"[Title/Abstract] OR "mild cognitive impairments"[Title/Abstract] OR "cognitive decline"[Title/Abstract] OR "cognitive declines"[Title/Abstract] OR "decline cognitive"[Title/Abstract] OR "declines cognitive"[Title/Abstract] OR "mental deterioration"[Title/Abstract] OR "deterioration mental"[Title/Abstract] OR (("deteriorate"[All Fields] OR "deteriorated"[All Fields] OR "deteriorates"[All Fields] OR "deteriorating"[All Fields] OR "Deterioration"[All Fields] OR "Deteriorations"[All Fields] OR "deteriorative"[All Fields]) AND "Mental"[Title/Abstract]) OR "mental deteriorations"[Title/Abstract])) 176 4:24:57  #6 ("Probiotics"[Mesh]) OR (Probiotic[Title/Abstract]) "Probiotics"[MeSH Terms] OR "Probiotic"[Title/Abstract] 36,630 4:13:01  #5 Probiotic[Title/Abstract] "Probiotic"[Title/Abstract] 26,815 4:12:30  #4 ("Cognitive Dysfunction"[Mesh]) OR (((((((((((((((((((((((((Cognitive Dysfunctions[Title/Abstract]) OR (Dysfunction, Cognitive[Title/Abstract])) OR (Dysfunctions, Cognitive[Title/Abstract])) OR (Cognitive Impairments[Title/Abstract])) OR (Cognitive Impairment[Title/Abstract])) OR (Impairment, Cognitive[Title/Abstract])) OR (Impairments, Cognitive[Title/Abstract])) OR (Cognitive Disorder[Title/Abstract])) OR (Cognitive Disorders[Title/Abstract])) OR (Disorder, Cognitive[Title/Abstract])) OR (Disorders, Cognitive[Title/Abstract])) OR (Mild Cognitive Impairment[Title/Abstract])) OR (Cognitive Impairment, Mild[Title/Abstract])) OR (Cognitive Impairments, Mild[Title/Abstract])) OR (Impairment, Mild Cognitive[Title/Abstract])) OR (Impairments, Mild Cognitive[Title/Abstract])) OR (Mild Cognitive Impairments[Title/Abstract])) OR (Cognitive Decline[Title/Abstract])) OR (Cognitive Declines[Title/Abstract])) OR (Decline, Cognitive[Title/Abstract])) OR (Declines, Cognitive[Title/Abstract])) OR (Mental Deterioration[Title/Abstract])) OR (Deterioration, Mental[Title/Abstract])) OR (Deteriorations, Mental[Title/Abstract])) OR (Mental Deteriorations[Title/Abstract])) "Cognitive Dysfunction"[MeSH Terms] OR ("cognitive dysfunctions"[Title/Abstract] OR "dysfunction cognitive"[Title/Abstract] OR "dysfunctions cognitive"[Title/Abstract] OR "cognitive impairments"[Title/Abstract] OR "cognitive impairment"[Title/Abstract] OR "impairment cognitive"[Title/Abstract] OR "impairments cognitive"[Title/Abstract] OR "cognitive disorder"[Title/Abstract] OR "cognitive disorders"[Title/Abstract] OR "disorder cognitive"[Title/Abstract] OR "disorders cognitive"[Title/Abstract] OR "mild cognitive impairment"[Title/Abstract] OR "cognitive impairment mild"[Title/Abstract] OR "cognitive impairments mild"[Title/Abstract] OR "impairment mild cognitive"[Title/Abstract] OR "impairments mild cognitive"[Title/Abstract] OR "mild cognitive impairments"[Title/Abstract] OR "cognitive decline"[Title/Abstract] OR "cognitive declines"[Title/Abstract] OR "decline cognitive"[Title/Abstract] OR "declines cognitive"[Title/Abstract] OR "mental deterioration"[Title/Abstract] OR "deterioration mental"[Title/Abstract] OR (("deteriorate"[All Fields] OR "deteriorated"[All Fields] OR "deteriorates"[All Fields] OR "deteriorating"[All Fields] OR "Deterioration"[All Fields] OR "Deteriorations"[All Fields] OR "deteriorative"[All Fields]) AND "Mental"[Title/Abstract]) OR "mental deteriorations"[Title/Abstract]) 139,335 4:08:39  #3 ((((((((((((((((((((((((Cognitive Dysfunctions[Title/Abstract]) OR (Dysfunction, Cognitive[Title/Abstract])) OR (Dysfunctions, Cognitive[Title/Abstract])) OR (Cognitive Impairments[Title/Abstract])) OR (Cognitive Impairment[Title/Abstract])) OR (Impairment, Cognitive[Title/Abstract])) OR (Impairments, Cognitive[Title/Abstract])) OR (Cognitive Disorder[Title/Abstract])) OR (Cognitive Disorders[Title/Abstract])) OR (Disorder, Cognitive[Title/Abstract])) OR (Disorders, Cognitive[Title/Abstract])) OR (Mild Cognitive Impairment[Title/Abstract])) OR (Cognitive Impairment, Mild[Title/Abstract])) OR (Cognitive Impairments, Mild[Title/Abstract])) OR (Impairment, Mild Cognitive[Title/Abstract])) OR (Impairments, Mild Cognitive[Title/Abstract])) OR (Mild Cognitive Impairments[Title/Abstract])) OR (Cognitive Decline[Title/Abstract])) OR (Cognitive Declines[Title/Abstract])) OR (Decline, Cognitive[Title/Abstract])) OR (Declines, Cognitive[Title/Abstract])) OR (Mental Deterioration[Title/Abstract])) OR (Deterioration, Mental[Title/Abstract])) OR (Deteriorations, Mental[Title/Abstract])) OR (Mental Deteriorations[Title/Abstract]) "cognitive dysfunctions"[Title/Abstract] OR "dysfunction cognitive"[Title/Abstract] OR "dysfunctions cognitive"[Title/Abstract] OR "cognitive impairments"[Title/Abstract] OR "cognitive impairment"[Title/Abstract] OR "impairment cognitive"[Title/Abstract] OR "impairments cognitive"[Title/Abstract] OR "cognitive disorder"[Title/Abstract] OR "cognitive disorders"[Title/Abstract] OR "disorder cognitive"[Title/Abstract] OR "disorders cognitive"[Title/Abstract] OR "mild cognitive impairment"[Title/Abstract] OR "cognitive impairment mild"[Title/Abstract] OR "cognitive impairments mild"[Title/Abstract] OR "impairment mild cognitive"[Title/Abstract] OR "impairments mild cognitive"[Title/Abstract] OR "mild cognitive impairments"[Title/Abstract] OR "cognitive decline"[Title/Abstract] OR "cognitive declines"[Title/Abstract] OR "decline cognitive"[Title/Abstract] OR "declines cognitive"[Title/Abstract] OR "mental deterioration"[Title/Abstract] OR "deterioration mental"[Title/Abstract] OR (("deteriorate"[All Fields] OR "deteriorated"[All Fields] OR "deteriorates"[All Fields] OR "deteriorating"[All Fields] OR "Deterioration"[All Fields] OR "Deteriorations"[All Fields] OR "deteriorative"[All Fields]) AND "Mental"[Title/Abstract]) OR "mental deteriorations"[Title/Abstract] 131,593 3:57:51  #2 "Cognitive Dysfunction"[Mesh] Most Recent "Cognitive Dysfunction"[MeSH Terms] 37,995 3:48:44  #1 "Probiotics"[Mesh] Most Recent "Probiotics"[MeSH Terms] 25,091 3:48:30  **(5)SinoMed**  序号 检索表达式 命中文献数 检索时间  1) "认知障碍"[不加权:扩展] 23219 2024-09-08 14:55:10.0  2) "认知功能障碍"[常用字段:智能] OR "术后认知并发症"[常用字段:智能] OR "认知功能下降"[常用字段:智能] OR "认知功能受损"[常用字段:智能] OR "神经认知障碍"[常用字段:智能] OR "神经系统并发症"[常用字段:智能] OR "脑功能障碍"[常用字段:智能] OR "ICU综合征"[常用字段:智能] OR "ICU精神病"[常用字段:智能] 803763 2024-09-08 15:02:58.0  3) "鼠李糖乳杆菌"[不加权:扩展] 4725 2024-09-08 15:03:51.0  4) "益生菌剂"[常用字段:智能] OR "有益菌"[常用字段:智能] OR "共生菌"[常用字段:智能] OR "益生元"[常用字段:智能] OR "微生态制剂"[常用字段:智能] OR "活菌制剂"[常用字段:智能] OR "有益微生物"[常用字段:智能] OR "益生菌"[常用字段:智能] 14231 2024-09-08 15:05:48.0  5) (#2) OR (#1) 803763 2024-09-08 15:06:27.0  6) (#4) OR (#3) 14231 2024-09-08 15:06:46.0  7) "随机对照试验"[不加权:扩展] 196514 2024-09-08 15:13:06.0  8) "随机对照"[常用字段:智能] OR "随机"[常用字段:智能] OR "RCT"[常用字段:智能] 1918508 2024-09-08 15:18:58.0  9) (#8) OR (#7) 1918723 2024-09-08 15:19:16.0  10) (#9) AND (#6) AND (#5) 198 2024-09-08 15:20:24.0  **(6)WeiPu Database**  [((((((((((题名或关键词=益生菌 OR 题名或关键词=probiotics) OR 题名或关键词=微生态制剂) OR 题名或关键词=活菌制剂) OR 题名或关键词=益生菌剂) OR 题名或关键词=益生素) OR 题名或关键词=有益菌) OR 题名或关键词=有益微生物) OR 题名或关键词=共生菌) OR 题名或关键词=益生元) AND ((((((((((((题名或关键词=认知障碍 OR 题名或关键词=cognition disorders) OR 题名或关键词=cognitive deficit) OR 题名或关键词=cognitive disorder) OR 题名或关键词=cognitive disorders) OR 题名或关键词=神经行为障碍) OR 题名或关键词=认知功能下降) OR 题名或关键词=认知功能受损) OR 题名或关键词=神经认知障碍) OR 题名或关键词=神经系统并发症) OR 题名或关键词=脑功能障碍) OR 题名或关键词=ICU综合征) OR 题名或关键词=ICU精神病))](https://http-qikan_cqvip_com.lib.henu.edu.cn/Qikan/search/index?LngMySearHistoryIdGuid=00f349a2-6589-4453-af94-ef6891085131&from=Qikan_Article_History)  **(7)Wanfang Database**  ((((((((题名或关键词=益生菌 OR 题名或关键词=微生态制剂) OR 题名或关键词=活菌制剂) OR 题名或关键词=益生菌剂) OR 题名或关键词=益生素) OR 题名或关键词=有益菌) OR 题名或关键词=有益微生物) OR 题名或关键词=益生元) AND ((((((((题名或关键词=认知功能障碍 ) OR 题名或关键词=认知功能下降) OR 题名或关键词=认知功能受损) OR 题名或关键词=神经认知障碍) OR 题名或关键词=神经系统并发症) OR 题名或关键词=脑功能障碍) OR 题名或关键词=ICU综合征) OR 题名或关键词=ICU精神病))  **(8)CNKI**  （主题：认知功能障碍）OR（主题：认知功能下降）OR（主题：认知功能受损）OR（主题：神经认知障碍）OR（主题：神经系统并发症）OR（主题：脑功能障碍）OR（主题：ICU综合征）OR（主题：ICU精神病）AND（主题：益生菌）OR（主题：益生菌剂）OR（主题：有益菌）OR（主题：共生菌）OR（主题：益生元）OR（主题：微生态制剂）OR（主题：活菌制剂）OR（主题：有益微生物） | Pages: 7-8 |
| Selection process | 8 | Two independent researchers meticulously screened the literature based on predefined inclusion and exclusion criteria, utilizing EndNote software to eliminate duplicates efficiently. In instances where disagreement arose during the screening process, a third researcher was consulted to engage in a collaborative discussion, ultimately determining the consensus on the final screening outcomes. When necessary, authors were reached out to procure crucial data that was integral to the analysis. | Page: 8 |
| Data collection process | 9 | Two reviewers independently extracted data from each report. Discrepancies were resolved by consensus or a third reviewer. Data were extracted using a standardized form. | Page: 8 |
| Data items | 10a | Primary outcome: cognitive function, measured using various scales. Secondary outcomes: changes in the number of intestinal flora, blood markers, etc. Only findings related to cognitive impairment were included in this study. | Pages: 8-9,11 |
|  | 10b | Participant characteristics: age, gender, disease duration, etc. Intervention characteristics: probiotic type, duration, type of scale used, etc.Missing or unclear information was assumed to be not available. | Pages: 8-9,11 |
| Study risk of bias assessment | 11 | For randomized controlled trials (RCTs), risk of bias was assessed using the Cochrane Handbook (5.1) risk of bias tool. Two independent reviewers assessed the risk of bias for each study. Discrepancies were resolved by discussion and consensus, and a third researcher was consulted if necessary. No automated tools were used in the risk of bias assessment process. | Page: 9 |
| Effect measures | 12 | The outcome measures reported in the included studies were continuous variables, and given the utilization of diverse measurement tools, the standard mean difference (SMD) was adopted as the effect size, with a corresponding 95% confidence interval (CI) calculated. | Pages: 9-10 |
| Synthesis methods | 13a | Tabulating the study intervention characteristics and comparing against the planned groups for each synthesis (item #5). | Pages: 6-7,11 |
|  | 13b | No lacking the necessary statistics in this study |  |
|  | 13c | This includes using tables, charts, or other visual tools to present the results of each study clearly. These tools can help readers more easily understand and compare the results between different studies. |  |
|  | 13d | A meta-analysis was conducted using Stata version 15.0 software. Heterogeneity across the studies was assessed based on both the P-value and the I² statistic. In cases where P > 0.1 and I² ≤ 50%, indicating the absence of significant heterogeneity, a fixed-effects model was chosen for analysis. Conversely, if P ≤ 0.1 and I² > 50%, suggesting substantial heterogeneity, a random-effects model was employed. Additionally, sensitivity analyses and subgroup analyses were conducted for variables that could potentially contribute to heterogeneity, accompanied by a bias assessment.The outcome measures reported in the included studies were continuous variables, and given the utilization of diverse measurement tools, the standard mean difference (SMD) was adopted as the effect size, with a corresponding 95% confidence interval (CI) calculated. | Pages: 9-10 |
|  | 13e | Reasons for heterogeneity were sought by subgroup analysis. | Page: 14 |
|  | 13f | A sensitivity analysis was conducted, involving the sequential exclusion of individual studies to compare changes in the combined outcomes. The analysis indicated that the changes in the results were insignificant, thereby demonstrating the robustness and stability of the study findings. | Page: 16 |
| Reporting bias assessment | 14 | Reporting bias was assessed by examining funnel plots for asymmetry and by testing for small study effects. | Pages: 10,16 |
| Certainty assessment | 15 | Evaluations were performed using the GRADE method according to randomized sequence, allocation concealment, subject/investigator blinding, outcome assessor blinding, data completeness, selective reporting, and other sources of bias. Evaluation results were expressed as “high risk,” “low risk,” or “unclear.” Studies were graded A if they fully met the assessment criteria, B if they met the criteria to some extent, and C if they did not meet the assessment criteria at all. | Page: 9 |
| **RESULTS** | | |  |
| Study selection | 16a | 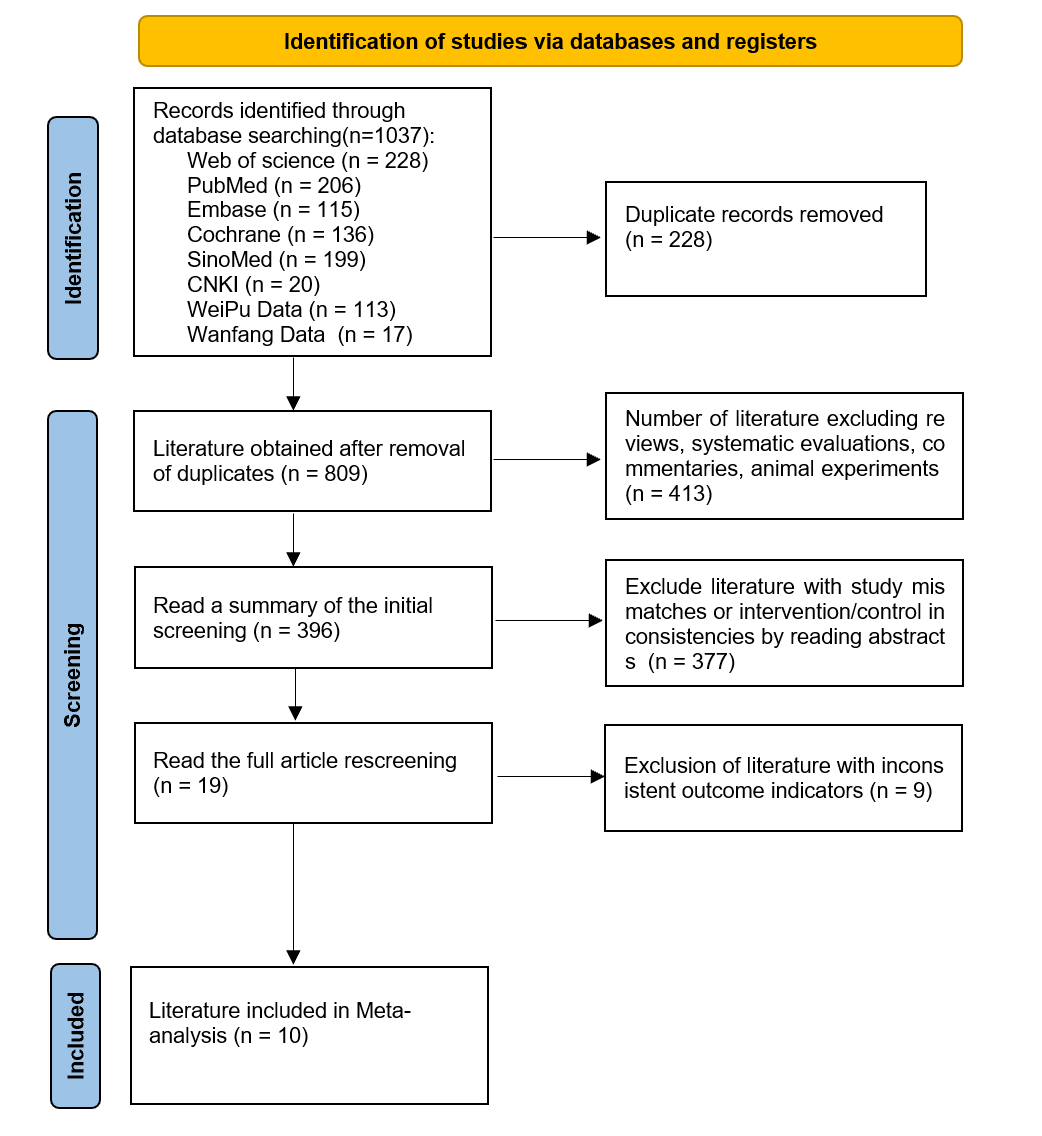 | Page: 10  (See Fig 1 in figure for details) |
|  | 16b | This study did not cite studies that may have appeared to meet the inclusion criteria but were excluded. |  |
| Study characteristics | 17 | 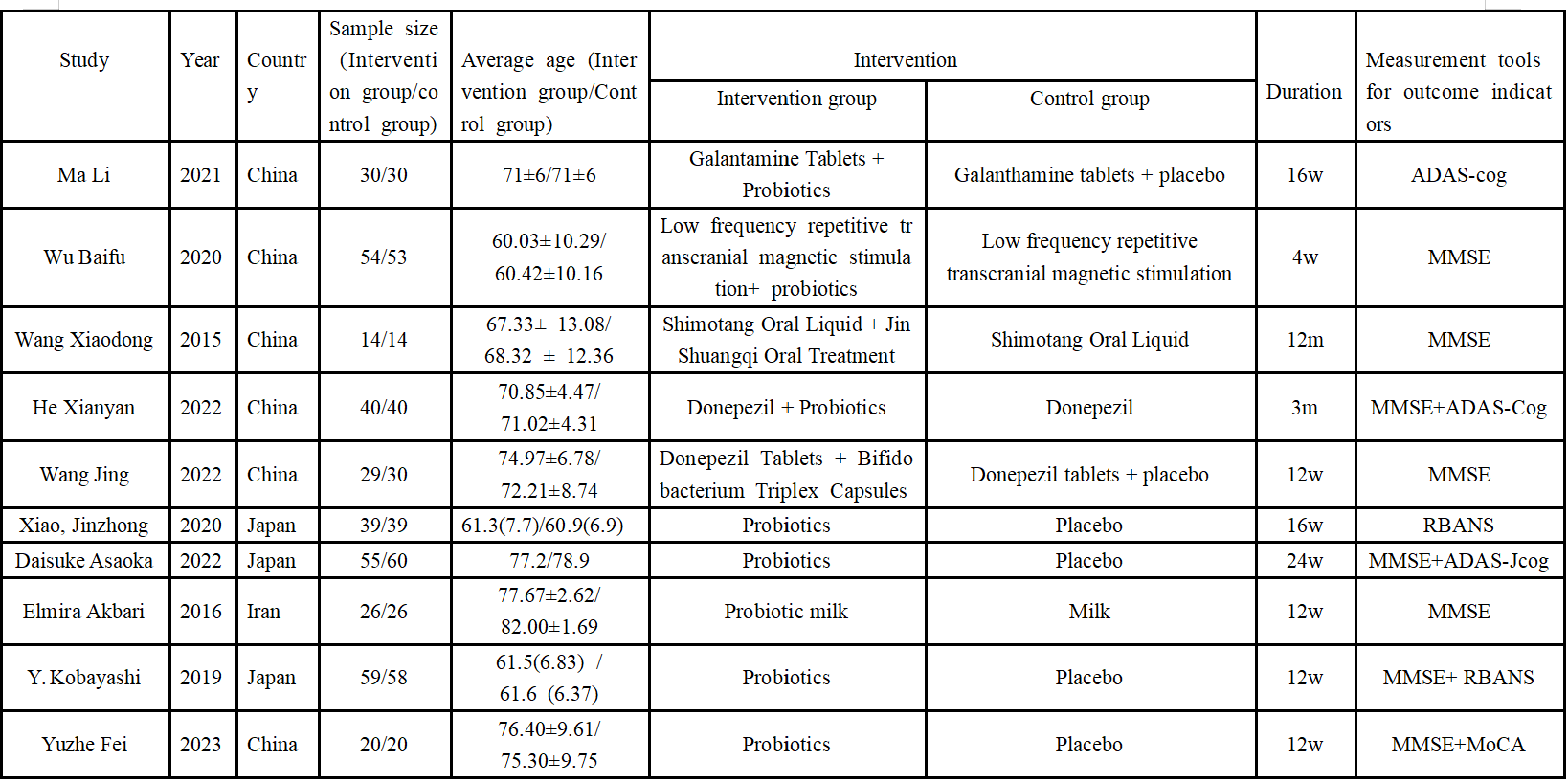 | Page: 11 |
| Risk of bias in studies | 18 | 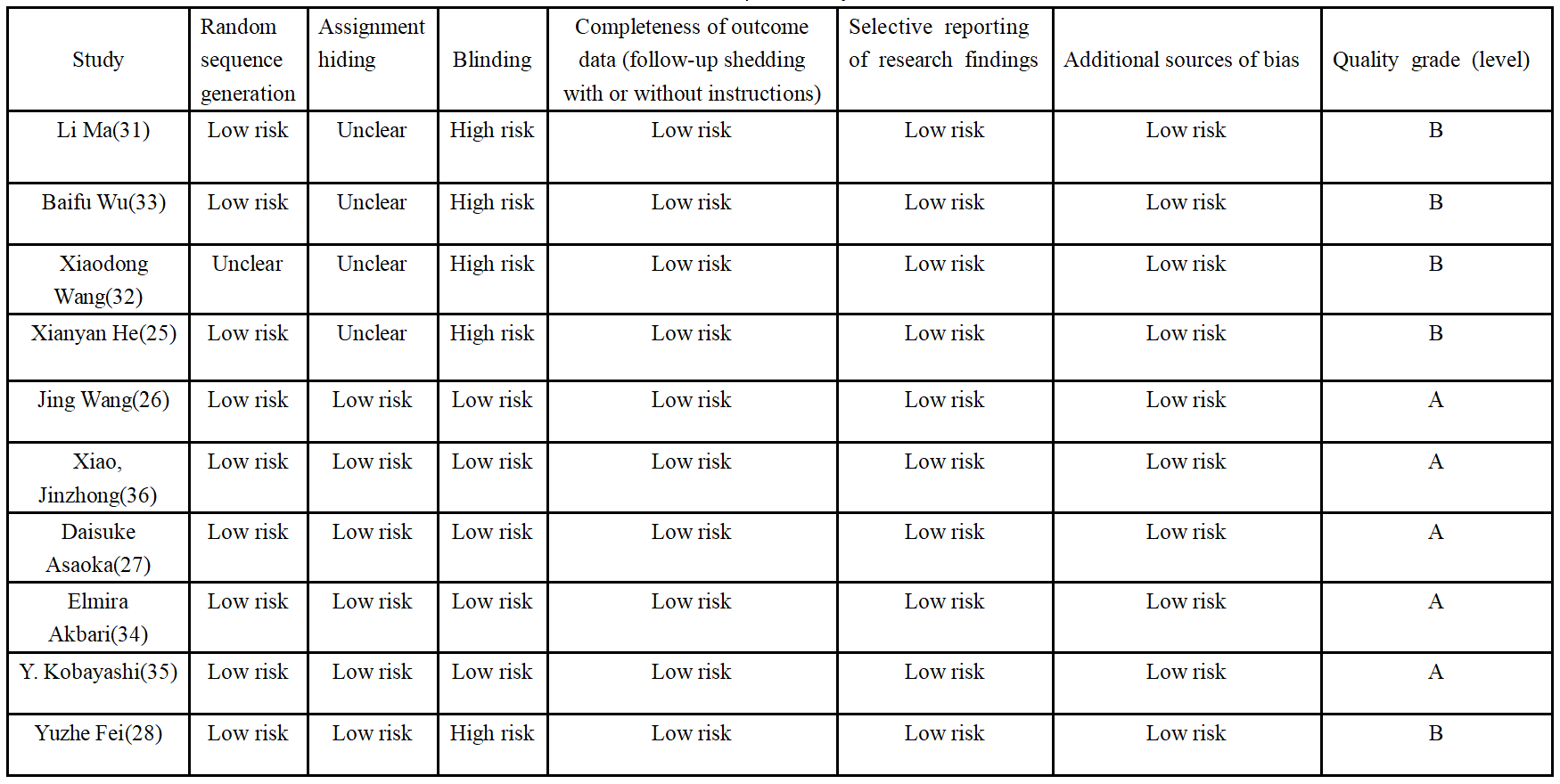 | Page: 13 |
| Results of individual studies | 19 | 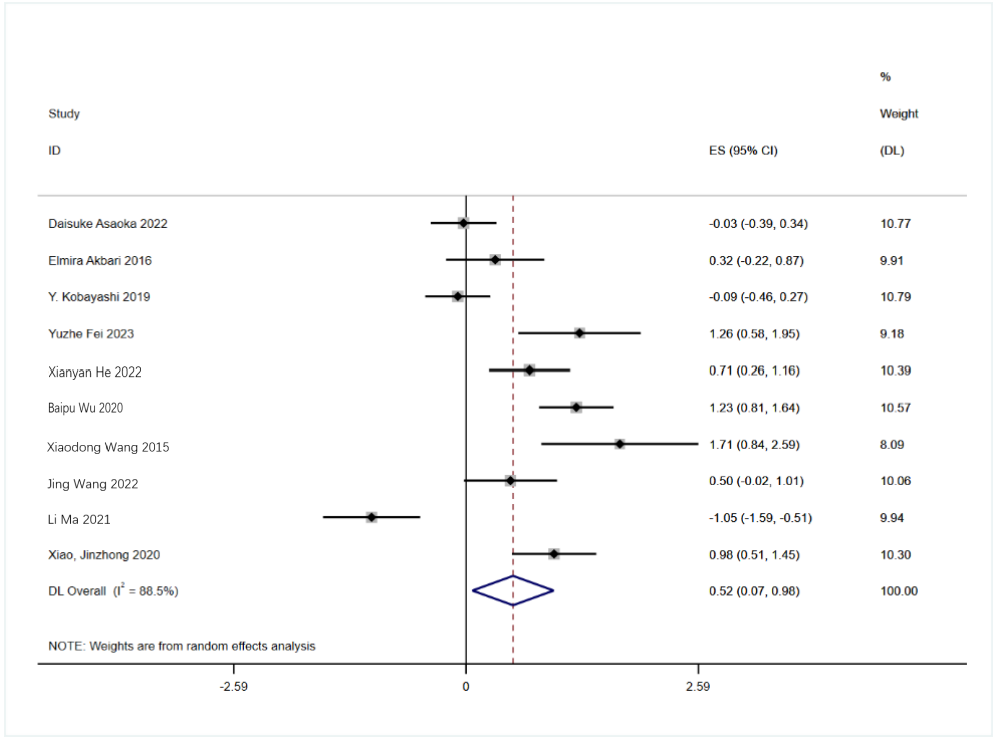 | Page: 14  (See Fig 2 in figure for details) |
| Results of syntheses | 20a | Among the ten studies included in this research, five were rated as quality A and five as quality B, all considered to be of high quality. Nine of these studies(25-28, 31, 33-36) detailed specific methods for generating randomized sequences. Six studies(26-28, 34-36) explicitly described the method used for allocation concealment in their text, while five studies(26, 27, 34-36) specified the blinding procedure for study participants or the individuals administering/measuring the intervention. The included literature comprehensively reported both primary and secondary outcome indicators | Page: 13 |
|  | 20b | 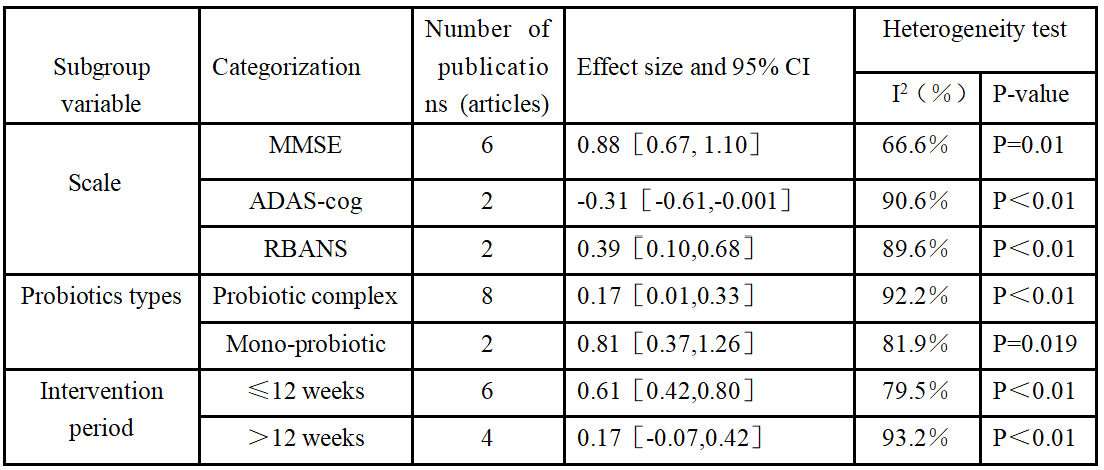 | Page: 16 |
|  | 20c | The potential sources of heterogeneity in this meta-analysis can be attributed to two factors: firstly, the subjective nature of cognitive functioning scales may contribute to increased heterogeneity in responses related to cognitive functioning effects. Secondly, the study participants hailed from diverse countries and utilized locally adapted scales to assess intervention effects. | Page: 17 |
|  | 20d | The literature included in the study were subjected to sensitivity analysis and compared the change in the combined results after excluding the literature piece by piece. The test found that the change in the results was not significant, indicating that the results of the current study were stable. | Page: 16 |
| Reporting biases | 21 | For each comprehensive assessment, we assessed the risk of bias due to missing outcomes (stemming from reporting bias). We used a funnel plot approach to detect potential publication bias. The results of the funnel plot analysis showed some asymmetry, which may imply the presence of publication bias in the study. Sensitivity analyses were performed and the changes in the combined results were compared after excluding literature on a piece-by-piece basis. The test found that the results did not change significantly, which indicates that the results of this study are stable. This was taken into account when interpreting these results and is stated accordingly in the conclusions. | Page: 16 |
| Certainty of evidence | 22 | Evidence for each outcome was assessed with certainty using the GRADE methodology. Evaluations were performed according to randomized sequence, allocation concealment, subject/investigator blinding, outcome assessor blinding, data completeness, selective reporting, and other sources of bias. The results were expressed as “high risk”, “low risk”, or “unclear”. Studies were graded A if they fully met the assessment criteria, B if they met the criteria to some extent, and C if they did not meet the assessment criteria at all. | Page: 9 |
| **DISCUSSION** | | |  |
| Discussion | 23a | In our study, we synthesized data from recent randomized controlled trials examining the use of probiotic and prebiotic supplements in patients with cognitive impairment. The results indicated that, compared to placebo or control treatments, probiotic supplementation exhibited statistically significant positive effects on cognitive function among these patients. Additionally, to delve deeper into the substantial heterogeneity observed among the included studies, we conducted a subgroup analysis. This analysis unveiled correlations between the magnitude of cognitive improvement and factors such as the type of probiotic strain (single versus combination), the duration of the intervention, and the specific cognitive assessment scale utilized. Collectively, these novel findings align with prior research documenting the neuroprotective benefits of probiotics and prebiotics in neurological disorders. | Pages: 17-21 |
|  | 23b | The current study is subject to several limitations. Firstly, the literature reviewed in this paper did not exclude the potential interference of other medications or forms of exercise, such as galantamine tablets, donepezil, moderate resistance training, and other factors. These medications and forms of exercise may exert a direct influence on gut microbiota composition and metabolic profiles, which could subsequently impact the gut-brain axis and related disorders. Therefore, it is imperative that future studies account for these variables. Secondly, heterogeneity may arise due to variations in probiotic supplement manufacturers. While probiotics as dietary supplements have been widely deemed safe for use based on prior research, the elderly population is particularly susceptible to serious adverse effects, including gastrointestinal discomfort, systemic infections, and skin issues, owing to their decreased immune function. In light of this, future studies must improve the reporting of potential adverse effects associated with probiotic supplements. | Page: 22 |
|  | 23c | First, quality assessment of included studies is based on predetermined criteria, but some subjective judgment may be involved in the process. Second, synthesizing results is challenging because of the heterogeneity of studies in terms of interventions and outcomes. | Page: 22 |
|  | 23d | The findings of this systematic review and meta-analysis have several implications for practice, policy, and future research. From a practice perspective, the results suggest that probiotics may be a promising adjunctive therapy for patients with cognitive impairment. However, given the limitations of the evidence, practitioners should exercise caution in recommending probiotics for this purpose and consider individual patient characteristics and needs. From a policy perspective, the results may inform the development of guidelines or recommendations regarding the use of probiotics in the management of cognitive impairment. Finally, future research should focus on conducting larger, well-designed, and rigorously conducted randomized controlled trials to further investigate the efficacy and safety of probiotics in patients with cognitive impairment. Additionally, studies should use consistent definitions and measurement tools for cognitive impairment to facilitate comparison and synthesis of findings across studies. | Page: 23 |
| **OTHER INFORMATION** | | |  |
| Registration and protocol | 24a | The systematic review titled "Efficacy of probiotics in patients with cognitive impairment: A systematic review and meta-analysis" was not registered. |  |
|  | 24b | A protocol for this systematic review was not prepared. |  |
|  | 24c | During the conduct of this systematic review, no amendments were made to the information provided at registration or in the protocol. All methods and objectives outlined in the protocol were adhered to throughout the review process. |  |
| Support | 25 | This study was supported by the Science and Technology Development of Henan Province in the year 2023 (232102310136). The funders were not associated with the conceptualization, design, implementation, or approval of this study. | Page: 24 |
| Competing interests | 26 | The authors have no relevant interests to declare. | Page: 24 |
| Availability of data, code and other materials | 27 | The data that support the findings of the study are available from the corresponding author upon reasonable request. | Page: 24 |

*From:*  Page MJ, McKenzie JE, Bossuyt PM, Boutron I, Hoffmann TC, Mulrow CD, et al. The PRISMA 2020 statement: an updated guideline for reporting systematic reviews. BMJ 2021;372:n71. doi: 10.1136/bmj.n71. This work is licensed under CC BY 4.0. To view a copy of this license, visit <https://creativecommons.org/licenses/by/4.0/>
