# Supplementary material for: Efficacy of probiotics in patients with cognitive impairment: A systematic review and meta-analysis
Source: PLoS One. 2025 May 2;20(5):e0321567. doi: 10.1371/journal.pone.0321567 (PMC12047807; doi:10.1371/journal.pone.0321567)
Supplement: S1 File — (DOCX) [file pone.0321567.s005.docx]

**Table 3. Quality evaluation of the literature**

| Study | Random sequence generation | Assignment hiding | Blinding | Completeness of outcome data (follow-up shedding with or without instructions) | Selective reporting of research findings | Additional sources of bias | Quality grade (level) |
| --- | --- | --- | --- | --- | --- | --- | --- |
| Daisuke Asaoka(27) | Low risk | Unclear | High risk | Low risk | Low risk | Low risk | B |
| Elmira Akbari(34) | Low risk | Unclear | High risk | Low risk | Low risk | Low risk | B |
| He Xianyan(25) | Unclear | Unclear | High risk | Low risk | Low risk | Low risk | B |
| Ma Li(31) | Low risk | Unclear | High risk | Low risk | Low risk | Low risk | B |
| Wang Jing(26) | Low risk | Low risk | Low risk | Low risk | Low risk | Low risk | A |
| Wu Baifu(33) | Low risk | Low risk | Low risk | Low risk | Low risk | Low risk | A |
| Wang Xiaodong(32) | Low risk | Low risk | Low risk | Low risk | Low risk | Low risk | A |
| Xiao, Jinzhong(36) | Low risk | Low risk | Low risk | Low risk | Low risk | Low risk | A |
| Y. Kobayashi(35) | Low risk | Low risk | Low risk | Low risk | Low risk | Low risk | A |
| Yuzhe Fei(28) | Low risk | Low risk | High risk | Low risk | Low risk | Low risk | B |
