# Supplementary material for: Efficacy of probiotics in patients with cognitive impairment: A systematic review and meta-analysis
Source: PLoS One. 2025 May 2;20(5):e0321567. doi: 10.1371/journal.pone.0321567 (PMC12047807; doi:10.1371/journal.pone.0321567)
Supplement: S2 File — (DOCX) [file pone.0321567.s006.docx]

**Search strategy**

**1：CNKI**

（主题：认知功能障碍）OR（主题：认知功能下降）OR（主题：认知功能受损）OR（主题：神经认知障碍）OR（主题：神经系统并发症）OR（主题：脑功能障碍）OR（主题：ICU综合征）OR（主题：ICU精神病）AND（主题：益生菌）OR（主题：益生菌剂）OR（主题：有益菌）OR（主题：共生菌）OR（主题：益生元）OR（主题：微生态制剂）OR（主题：活菌制剂）OR（主题：有益微生物）

**2：Wanfang Database**

((((((((题名或关键词=益生菌 OR 题名或关键词=微生态制剂) OR 题名或关键词=活菌制剂) OR 题名或关键词=益生菌剂) OR 题名或关键词=益生素) OR 题名或关键词=有益菌) OR 题名或关键词=有益微生物) OR 题名或关键词=益生元) AND ((((((((题名或关键词=认知功能障碍 ) OR 题名或关键词=认知功能下降) OR 题名或关键词=认知功能受损) OR 题名或关键词=神经认知障碍) OR 题名或关键词=神经系统并发症) OR 题名或关键词=脑功能障碍) OR 题名或关键词=ICU综合征) OR 题名或关键词=ICU精神病))

**3：WeiPu Database**

[((((((((((题名或关键词=益生菌 OR 题名或关键词=probiotics) OR 题名或关键词=微生态制剂) OR 题名或关键词=活菌制剂) OR 题名或关键词=益生菌剂) OR 题名或关键词=益生素) OR 题名或关键词=有益菌) OR 题名或关键词=有益微生物) OR 题名或关键词=共生菌) OR 题名或关键词=益生元) AND ((((((((((((题名或关键词=认知障碍 OR 题名或关键词=cognition disorders) OR 题名或关键词=cognitive deficit) OR 题名或关键词=cognitive disorder) OR 题名或关键词=cognitive disorders) OR 题名或关键词=神经行为障碍) OR 题名或关键词=认知功能下降) OR 题名或关键词=认知功能受损) OR 题名或关键词=神经认知障碍) OR 题名或关键词=神经系统并发症) OR 题名或关键词=脑功能障碍) OR 题名或关键词=ICU综合征) OR 题名或关键词=ICU精神病))](https://http-qikan_cqvip_com.lib.henu.edu.cn/Qikan/search/index?LngMySearHistoryIdGuid=00f349a2-6589-4453-af94-ef6891085131&from=Qikan_Article_History)

**4：SinoMed**

序号 检索表达式 命中文献数 检索时间

1) "认知障碍"[不加权:扩展] 23219 2024-09-08 14:55:10.0

2) "认知功能障碍"[常用字段:智能] OR "术后认知并发症"[常用字段:智能] OR "认知功能下降"[常用字段:智能] OR "认知功能受损"[常用字段:智能] OR "神经认知障碍"[常用字段:智能] OR "神经系统并发症"[常用字段:智能] OR "脑功能障碍"[常用字段:智能] OR "ICU综合征"[常用字段:智能] OR "ICU精神病"[常用字段:智能] 803763 2024-09-08 15:02:58.0

3) "鼠李糖乳杆菌"[不加权:扩展] 4725 2024-09-08 15:03:51.0

4) "益生菌剂"[常用字段:智能] OR "有益菌"[常用字段:智能] OR "共生菌"[常用字段:智能] OR "益生元"[常用字段:智能] OR "微生态制剂"[常用字段:智能] OR "活菌制剂"[常用字段:智能] OR "有益微生物"[常用字段:智能] OR "益生菌"[常用字段:智能] 14231 2024-09-08 15:05:48.0

5) (#2) OR (#1) 803763 2024-09-08 15:06:27.0

6) (#4) OR (#3) 14231 2024-09-08 15:06:46.0

7) "随机对照试验"[不加权:扩展] 196514 2024-09-08 15:13:06.0

8) "随机对照"[常用字段:智能] OR "随机"[常用字段:智能] OR "RCT"[常用字段:智能] 1918508 2024-09-08 15:18:58.0

9) (#8) OR (#7) 1918723 2024-09-08 15:19:16.0

10) (#9) AND (#6) AND (#5) 198 2024-09-08 15:20:24.0

**5：PubMed**

#7 (("Probiotics"[Mesh]) OR (Probiotic[Title/Abstract])) AND (("Cognitive Dysfunction"[Mesh]) OR (((((((((((((((((((((((((Cognitive Dysfunctions[Title/Abstract]) OR (Dysfunction, Cognitive[Title/Abstract])) OR (Dysfunctions, Cognitive[Title/Abstract])) OR (Cognitive Impairments[Title/Abstract])) OR (Cognitive Impairment[Title/Abstract])) OR (Impairment, Cognitive[Title/Abstract])) OR (Impairments, Cognitive[Title/Abstract])) OR (Cognitive Disorder[Title/Abstract])) OR (Cognitive Disorders[Title/Abstract])) OR (Disorder, Cognitive[Title/Abstract])) OR (Disorders, Cognitive[Title/Abstract])) OR (Mild Cognitive Impairment[Title/Abstract])) OR (Cognitive Impairment, Mild[Title/Abstract])) OR (Cognitive Impairments, Mild[Title/Abstract])) OR (Impairment, Mild Cognitive[Title/Abstract])) OR (Impairments, Mild Cognitive[Title/Abstract])) OR (Mild Cognitive Impairments[Title/Abstract])) OR (Cognitive Decline[Title/Abstract])) OR (Cognitive Declines[Title/Abstract])) OR (Decline, Cognitive[Title/Abstract])) OR (Declines, Cognitive[Title/Abstract])) OR (Mental Deterioration[Title/Abstract])) OR (Deterioration, Mental[Title/Abstract])) OR (Deteriorations, Mental[Title/Abstract])) OR (Mental Deteriorations[Title/Abstract]))) ("Probiotics"[MeSH Terms] OR "Probiotic"[Title/Abstract]) AND ("Cognitive Dysfunction"[MeSH Terms] OR ("cognitive dysfunctions"[Title/Abstract] OR "dysfunction cognitive"[Title/Abstract] OR "dysfunctions cognitive"[Title/Abstract] OR "cognitive impairments"[Title/Abstract] OR "cognitive impairment"[Title/Abstract] OR "impairment cognitive"[Title/Abstract] OR "impairments cognitive"[Title/Abstract] OR "cognitive disorder"[Title/Abstract] OR "cognitive disorders"[Title/Abstract] OR "disorder cognitive"[Title/Abstract] OR "disorders cognitive"[Title/Abstract] OR "mild cognitive impairment"[Title/Abstract] OR "cognitive impairment mild"[Title/Abstract] OR "cognitive impairments mild"[Title/Abstract] OR "impairment mild cognitive"[Title/Abstract] OR "impairments mild cognitive"[Title/Abstract] OR "mild cognitive impairments"[Title/Abstract] OR "cognitive decline"[Title/Abstract] OR "cognitive declines"[Title/Abstract] OR "decline cognitive"[Title/Abstract] OR "declines cognitive"[Title/Abstract] OR "mental deterioration"[Title/Abstract] OR "deterioration mental"[Title/Abstract] OR (("deteriorate"[All Fields] OR "deteriorated"[All Fields] OR "deteriorates"[All Fields] OR "deteriorating"[All Fields] OR "Deterioration"[All Fields] OR "Deteriorations"[All Fields] OR "deteriorative"[All Fields]) AND "Mental"[Title/Abstract]) OR "mental deteriorations"[Title/Abstract])) 176 4:24:57

#6 ("Probiotics"[Mesh]) OR (Probiotic[Title/Abstract]) "Probiotics"[MeSH Terms] OR "Probiotic"[Title/Abstract] 36,630 4:13:01

#5 Probiotic[Title/Abstract] "Probiotic"[Title/Abstract] 26,815 4:12:30

#4 ("Cognitive Dysfunction"[Mesh]) OR (((((((((((((((((((((((((Cognitive Dysfunctions[Title/Abstract]) OR (Dysfunction, Cognitive[Title/Abstract])) OR (Dysfunctions, Cognitive[Title/Abstract])) OR (Cognitive Impairments[Title/Abstract])) OR (Cognitive Impairment[Title/Abstract])) OR (Impairment, Cognitive[Title/Abstract])) OR (Impairments, Cognitive[Title/Abstract])) OR (Cognitive Disorder[Title/Abstract])) OR (Cognitive Disorders[Title/Abstract])) OR (Disorder, Cognitive[Title/Abstract])) OR (Disorders, Cognitive[Title/Abstract])) OR (Mild Cognitive Impairment[Title/Abstract])) OR (Cognitive Impairment, Mild[Title/Abstract])) OR (Cognitive Impairments, Mild[Title/Abstract])) OR (Impairment, Mild Cognitive[Title/Abstract])) OR (Impairments, Mild Cognitive[Title/Abstract])) OR (Mild Cognitive Impairments[Title/Abstract])) OR (Cognitive Decline[Title/Abstract])) OR (Cognitive Declines[Title/Abstract])) OR (Decline, Cognitive[Title/Abstract])) OR (Declines, Cognitive[Title/Abstract])) OR (Mental Deterioration[Title/Abstract])) OR (Deterioration, Mental[Title/Abstract])) OR (Deteriorations, Mental[Title/Abstract])) OR (Mental Deteriorations[Title/Abstract])) "Cognitive Dysfunction"[MeSH Terms] OR ("cognitive dysfunctions"[Title/Abstract] OR "dysfunction cognitive"[Title/Abstract] OR "dysfunctions cognitive"[Title/Abstract] OR "cognitive impairments"[Title/Abstract] OR "cognitive impairment"[Title/Abstract] OR "impairment cognitive"[Title/Abstract] OR "impairments cognitive"[Title/Abstract] OR "cognitive disorder"[Title/Abstract] OR "cognitive disorders"[Title/Abstract] OR "disorder cognitive"[Title/Abstract] OR "disorders cognitive"[Title/Abstract] OR "mild cognitive impairment"[Title/Abstract] OR "cognitive impairment mild"[Title/Abstract] OR "cognitive impairments mild"[Title/Abstract] OR "impairment mild cognitive"[Title/Abstract] OR "impairments mild cognitive"[Title/Abstract] OR "mild cognitive impairments"[Title/Abstract] OR "cognitive decline"[Title/Abstract] OR "cognitive declines"[Title/Abstract] OR "decline cognitive"[Title/Abstract] OR "declines cognitive"[Title/Abstract] OR "mental deterioration"[Title/Abstract] OR "deterioration mental"[Title/Abstract] OR (("deteriorate"[All Fields] OR "deteriorated"[All Fields] OR "deteriorates"[All Fields] OR "deteriorating"[All Fields] OR "Deterioration"[All Fields] OR "Deteriorations"[All Fields] OR "deteriorative"[All Fields]) AND "Mental"[Title/Abstract]) OR "mental deteriorations"[Title/Abstract]) 139,335 4:08:39

#3 ((((((((((((((((((((((((Cognitive Dysfunctions[Title/Abstract]) OR (Dysfunction, Cognitive[Title/Abstract])) OR (Dysfunctions, Cognitive[Title/Abstract])) OR (Cognitive Impairments[Title/Abstract])) OR (Cognitive Impairment[Title/Abstract])) OR (Impairment, Cognitive[Title/Abstract])) OR (Impairments, Cognitive[Title/Abstract])) OR (Cognitive Disorder[Title/Abstract])) OR (Cognitive Disorders[Title/Abstract])) OR (Disorder, Cognitive[Title/Abstract])) OR (Disorders, Cognitive[Title/Abstract])) OR (Mild Cognitive Impairment[Title/Abstract])) OR (Cognitive Impairment, Mild[Title/Abstract])) OR (Cognitive Impairments, Mild[Title/Abstract])) OR (Impairment, Mild Cognitive[Title/Abstract])) OR (Impairments, Mild Cognitive[Title/Abstract])) OR (Mild Cognitive Impairments[Title/Abstract])) OR (Cognitive Decline[Title/Abstract])) OR (Cognitive Declines[Title/Abstract])) OR (Decline, Cognitive[Title/Abstract])) OR (Declines, Cognitive[Title/Abstract])) OR (Mental Deterioration[Title/Abstract])) OR (Deterioration, Mental[Title/Abstract])) OR (Deteriorations, Mental[Title/Abstract])) OR (Mental Deteriorations[Title/Abstract]) "cognitive dysfunctions"[Title/Abstract] OR "dysfunction cognitive"[Title/Abstract] OR "dysfunctions cognitive"[Title/Abstract] OR "cognitive impairments"[Title/Abstract] OR "cognitive impairment"[Title/Abstract] OR "impairment cognitive"[Title/Abstract] OR "impairments cognitive"[Title/Abstract] OR "cognitive disorder"[Title/Abstract] OR "cognitive disorders"[Title/Abstract] OR "disorder cognitive"[Title/Abstract] OR "disorders cognitive"[Title/Abstract] OR "mild cognitive impairment"[Title/Abstract] OR "cognitive impairment mild"[Title/Abstract] OR "cognitive impairments mild"[Title/Abstract] OR "impairment mild cognitive"[Title/Abstract] OR "impairments mild cognitive"[Title/Abstract] OR "mild cognitive impairments"[Title/Abstract] OR "cognitive decline"[Title/Abstract] OR "cognitive declines"[Title/Abstract] OR "decline cognitive"[Title/Abstract] OR "declines cognitive"[Title/Abstract] OR "mental deterioration"[Title/Abstract] OR "deterioration mental"[Title/Abstract] OR (("deteriorate"[All Fields] OR "deteriorated"[All Fields] OR "deteriorates"[All Fields] OR "deteriorating"[All Fields] OR "Deterioration"[All Fields] OR "Deteriorations"[All Fields] OR "deteriorative"[All Fields]) AND "Mental"[Title/Abstract]) OR "mental deteriorations"[Title/Abstract] 131,593 3:57:51

#2 "Cognitive Dysfunction"[Mesh] Most Recent "Cognitive Dysfunction"[MeSH Terms] 37,995 3:48:44

#1 "Probiotics"[Mesh] Most Recent "Probiotics"[MeSH Terms] 25,091 3:48:30

**6：Web of Science**

# Web of Science Search Strategy (v0.1)

# Database: All Databases

# Entitlements:

- WOS: 1985 to 2024

- BIOSIS: 2011 to 2024

- CSCD: 1989 to 2024

- KJD: 1980 to 2024

- MEDLINE: 1950 to 2024

- PPRN: 1991 to 2024

- PQDT: 1637 to 2024

- SCIELO: 2002 to 2024

# Searches:

1: Cognitive Dysfunction (Topic) OR Cognitive Dysfunctions (Topic) OR Dysfunction, Cognitive

(Topic) OR Dysfunctions, Cognitive (Topic) OR Cognitive Impairments (Topic) OR Cognitive

Impairment (Topic) OR Impairment, Cognitive (Topic) OR Impairments, Cognitive (Topic) OR

Cognitive Disorder (Topic) OR Cognitive Disorders (Topic) OR Disorder, Cognitive (Topic) OR

Disorders, Cognitive (Topic) OR Mild Cognitive Impairment (Topic) OR Cognitive Impairment,

Mild (Topic) OR Cognitive Impairments, Mild (Topic) OR Impairment, Mild Cognitive (Topic) OR Impairments, Mild Cognitive (Topic) OR Mild Cognitive Impairments (Topic) OR Cognitive

Decline (Topic) OR Cognitive Declines (Topic) OR Decline, Cognitive (Topic) OR Declines,

Cognitive (Topic) OR Mental Deterioration (Topic) OR Deterioration, Mental (Topic) OR

Deteriorations, Mental (Topic) OR Mental Deteriorations (Topic) and Preprint Citation Index

(Exclude – Database) Date Run: Fri Sep 09 2024 14:25:23 GMT+0800

(GMT+08:00) Results: 526454

2: Probiotics (Topic) OR Probiotic (Topic) and Preprint Citation Index (Exclude –

Database) Date Run: Fri Sep 09 2024 14:26:26 GMT+0800 (GMT+08:00) Results: 82020

3: ((((TS=(randomized controlled trial)) OR TS=(randomized)) OR TS=(placebo)) OR

TS=(randomised)) OR TS=(random) and Preprint Citation Index (Exclude – Database) Date

Run: Fri Sep 09 2024 14:32:44 GMT+0800 (GMT+08:00) Results: 2742637

4: #1 AND #2 AND #3 and Preprint Citation Index (Exclude – Database) Date Run: Fri Sep

09 2024 14:34:51 GMT+0800 (GMT+08:00) Results: 222

**7：Embase**

Embase

Session Results

.......................................................

No. Query Results Results Date

#8. #3 AND #6 AND #7 99 Sep 2024

#7. 'randomized controlled trial':ab,ti OR 1,159,505 Sep 2024

'randomized':ab,ti OR 'placebo':ab,ti

#6. #4 OR #5 63,736 Sep 2024

#5. 'probiotic':ab,ti OR 'probiotics':ab,ti 47,356 Sep 2024

#4. 'probiotic agent'/exp 54,599 Sep 2024

#3. #1 OR #2 660,378 Sep 2024

#2. 'cognitive dysfunctions':ab,ti OR 'dysfunction, 183,972 Sep 2024

cognitive':ab,ti OR 'dysfunctions,

cognitive':ab,ti OR 'cognitive impairments':ab,ti

OR 'cognitive impairment':ab,ti OR 'impairment,

cognitive':ab,ti OR 'impairments,

cognitive':ab,ti OR 'cognitive disorder':ab,ti OR

'cognitive disorders':ab,ti OR 'disorder,

cognitive':ab,ti OR 'disorders, cognitive':ab,ti

OR 'mild cognitive impairment':ab,ti OR

'cognitive impairment, mild':ab,ti OR 'cognitive

impairments, mild':ab,ti OR 'impairment, mild

cognitive':ab,ti OR 'impairments, mild

cognitive':ab,ti OR 'mild cognitive

impairments':ab,ti OR 'cognitive decline':ab,ti

OR 'cognitive declines':ab,ti OR 'decline,

cognitive':ab,ti OR 'declines, cognitive':ab,ti

OR 'mental deterioration':ab,ti OR

'deterioration, mental':ab,ti OR 'deteriorations,

mental':ab,ti OR 'mental deteriorations':ab,ti

#1. 'cognitive defect'/exp 628,449 Sep 2024

**8：Cochrane Library**

Search Name: 检索历史

Date Run: 07/09/2024 22:16:52

Comment:

ID Search Hits

#1 MeSH descriptor: [Cognitive Dysfunction] explode all trees 3159

#2 (Cognitive Dysfunctions):ti,ab,kw OR (Dysfunction, Cognitive):ti,ab,kw OR (Dysfunctions, Cognitive):ti,ab,kw OR (Cognitive Impairments):ti,ab,kw OR (Cognitive Impairment):ti,ab,kw OR (Impairment, Cognitive):ti,ab,kw OR (Impairments, Cognitive):ti,ab,kw OR (Cognitive Disorder):ti,ab,kw OR (Cognitive Disorders):ti,ab,kw OR (Disorder, Cognitive):ti,ab,kw OR (Disorders, Cognitive):ti,ab,kw OR (Mild Cognitive Impairment):ti,ab,kw OR (Cognitive Impairment, Mild):ti,ab,kw OR (Cognitive Impairments, Mild):ti,ab,kw OR (Impairment, Mild Cognitive):ti,ab,kw OR (Impairments, Mild Cognitive):ti,ab,kw OR (Mild Cognitive Impairments):ti,ab,kw OR (Cognitive Decline):ti,ab,kw OR (Cognitive Declines):ti,ab,kw OR (Decline, Cognitive):ti,ab,kw OR (Declines, Cognitive):ti,ab,kw OR (Mental Deterioration):ti,ab,kw OR (Deterioration, Mental):ti,ab,kw OR (Deteriorations, Mental):ti,ab,kw OR (Mental Deteriorations):ti,ab,kw 53629

#3 #1 OR #2 53640

#4 MeSH descriptor: [Probiotics] explode all trees 3122

#5 (Probiotic):ti,ab,kw 7159

#6 #4 OR #5 8040

#7 #3 AND #6 123
