# Supplementary material for: Efficacy of probiotics in patients with cognitive impairment: A systematic review and meta-analysis
Source: PLoS One. 2025 May 2;20(5):e0321567. doi: 10.1371/journal.pone.0321567 (PMC12047807; doi:10.1371/journal.pone.0321567)
Supplement: S3 File — (DOCX) [file pone.0321567.s007.docx]

**Fig 1. PRISMA flow chart for the study selection process**


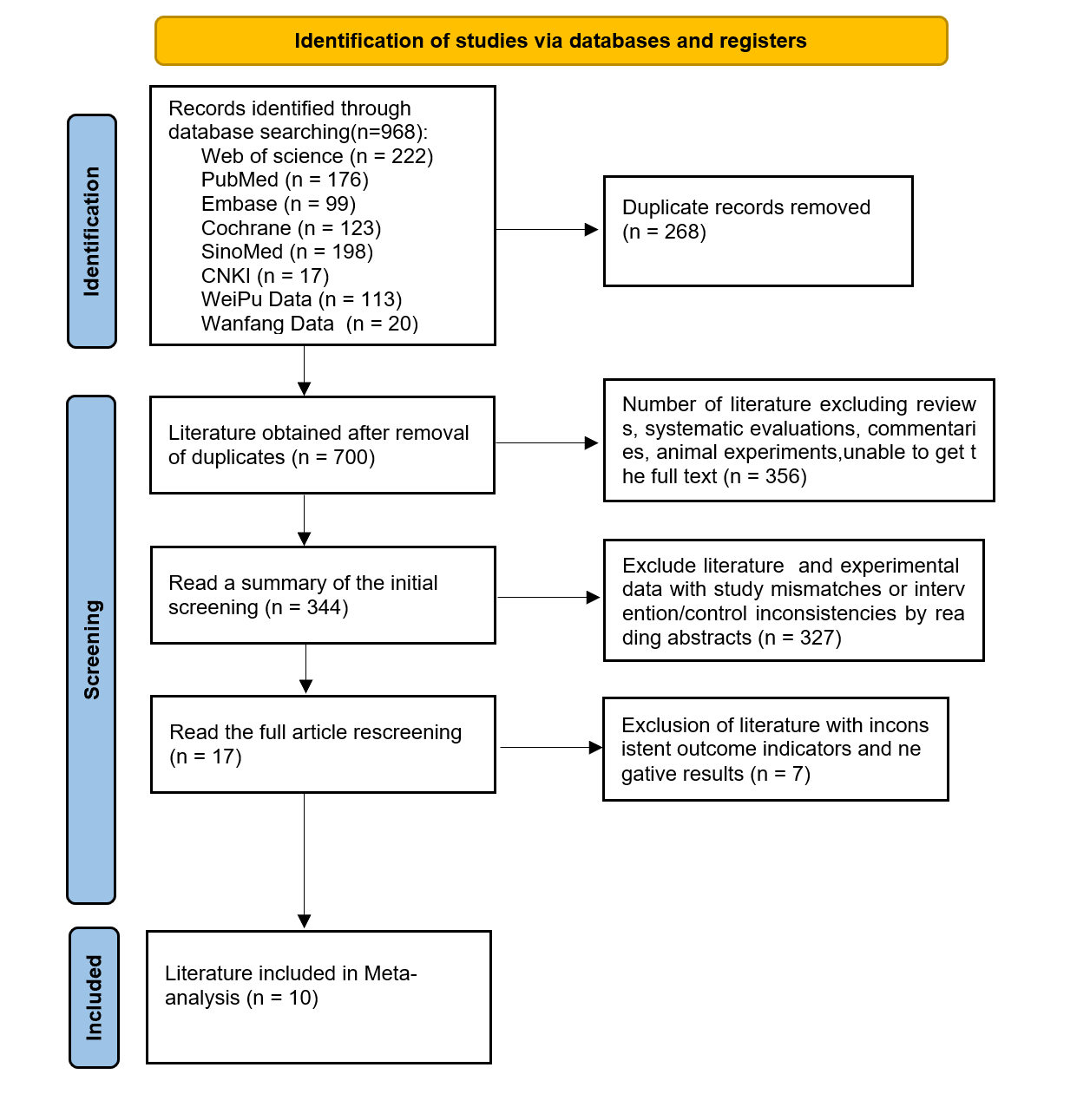


**Table 2. Basic characteristics and quality evaluation of the included literature**

| Study | Year | Country | Sample size (Intervention group/control group) | Average age (Intervention group/Control group) | Intervention | | Duration | Measurement tools for outcome indicators |
| --- | --- | --- | --- | --- | --- | --- | --- | --- |
|  |  |  |  |  | Intervention group | Control group |  |  |
| Daisuke Asaoka | 2022 | Japan | 55/60 | 77.2/78.9 | Probiotics | Placebo | 24w | MMSE+ADAS-Jcog |
| Elmira Akbari | 2016 | Iran | 26/26 | 77.67±2.62/  82.00±1.69 | Probiotic milk | Milk | 12w | MMSE |
| He Xianyan | 2022 | China | 40/40 | 70.85±4.47/  71.02±4.31 | Donepezil + Probiotics | Donepezil | 3m | MMSE+ADAS-Cog |
| Ma Li | 2021 | China | 30/30 | 71±6/71±6 | Galantamine Tablets + Probiotics | Galanthamine tablets + placebo | 16w | ADAS-cog |
| Wu Baifu | 2020 | China | 54/53 | 60.03±10.29/  60.42±10.16 | Low frequency repetitive transcranial magnetic stimulation+ probiotics | Low frequency repetitive transcranial magnetic stimulation | 4w | MMSE |
| Wang Xiaodong | 2015 | China | 14/14 | 67.33± 13.08/  68.32 ± 12.36 | Shimotang Oral Liquid + Jin Shuangqi Oral Treatment | Shimotang Oral Liquid | 12m | MMSE |
| Wang Jing | 2022 | China | 29/30 | 74.97±6.78/  72.21±8.74 | Donepezil Tablets + Bifidobacterium Triplex Capsules | Donepezil tablets + placebo | 12w | MMSE |
| Xiao, Jinzhong | 2020 | Japan | 39/39 | 61.3(7.7)/60.9(6.9) | Probiotics | Placebo | 16w | RBANS |
| Y. Kobayashi | 2019 | Japan | 59/58 | 61.5(6.83) /  61.6 (6.37) | Probiotics | Placebo | 12w | MMSE+ RBANS |
| Yuzhe Fei | 2023 | China | 20/20 | 76.40±9.61/  75.30±9.75 | Probiotics | Placebo | 12w | MMSE+MoCA |
