# Supplementary material for: Efficacy of probiotics in patients with cognitive impairment: A systematic review and meta-analysis
Source: PLoS One. 2025 May 2;20(5):e0321567. doi: 10.1371/journal.pone.0321567 (PMC12047807; doi:10.1371/journal.pone.0321567)
Supplement: S4 File — (DOCX) [file pone.0321567.s008.docx]

**Fig 2. Forest plot of the overall effect of probiotics on cognition in a random-effects model**


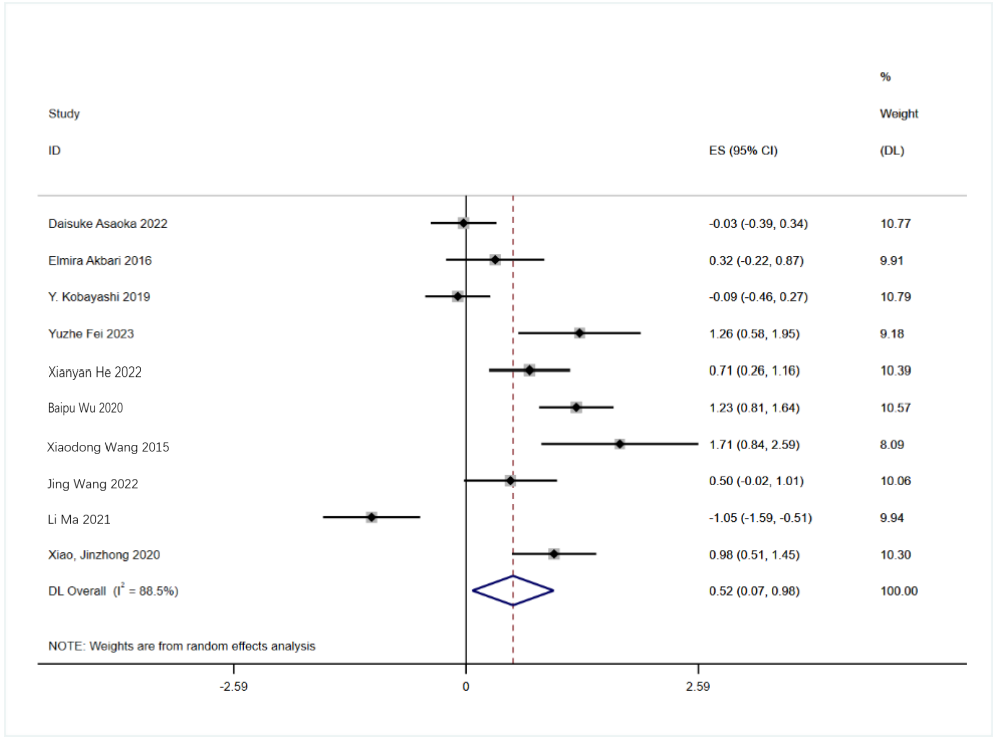


**Table 4. Subgroup analysis results**

| Subgroup variable | Categorization | Number of publications (articles) | Effect size and 95% CI | Heterogeneity test | |
| --- | --- | --- | --- | --- | --- |
|  |  |  |  | I^2^（％） | P-value |
| Scale | MMSE | 6 | 0.88［0.67, 1.10］ | 66.6％ | P=0.01 |
|  | ADAS-cog | 2 | -0.31［-0.61,-0.001］ | 90.6％ | P＜0.01 |
|  | RBANS | 2 | 0.39［0.10,0.68］ | 89.6％ | P＜0.01 |
| Probiotics types | Probiotic complex | 8 | 0.17［0.01,0.33］ | 92.2％ | P＜0.01 |
|  | Mono-probiotic | 2 | 0.81［0.37,1.26］ | 81.9％ | P=0.019 |
| Intervention period | ≤12 weeks | 6 | 0.61［0.42,0.80］ | 79.5％ | P＜0.01 |
|  | ＞12 weeks | 4 | 0.17［-0.07,0.42］ | 93.2％ | P＜0.01 |

**Fig 3. Funnel plot**

**
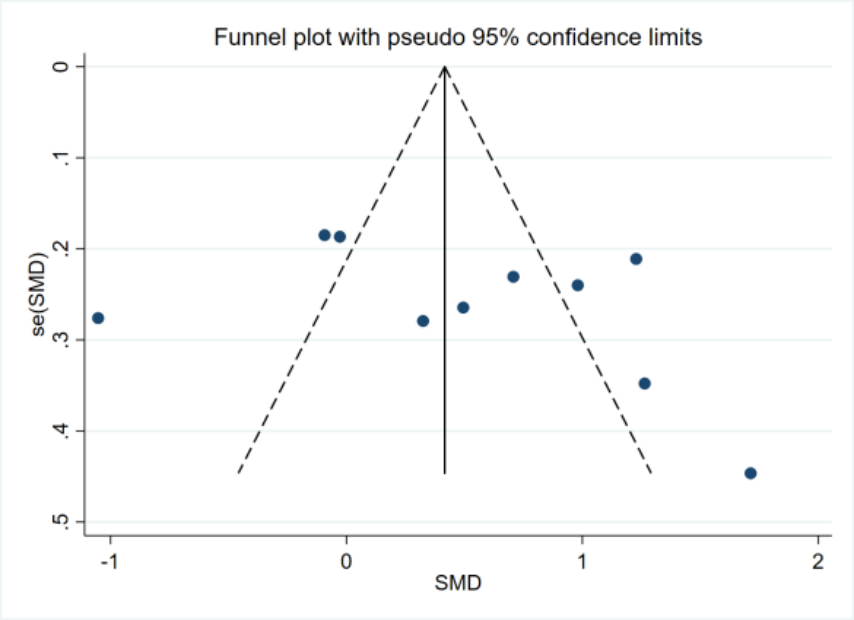
**

**Fig 4. Sensitivity analysis**

**
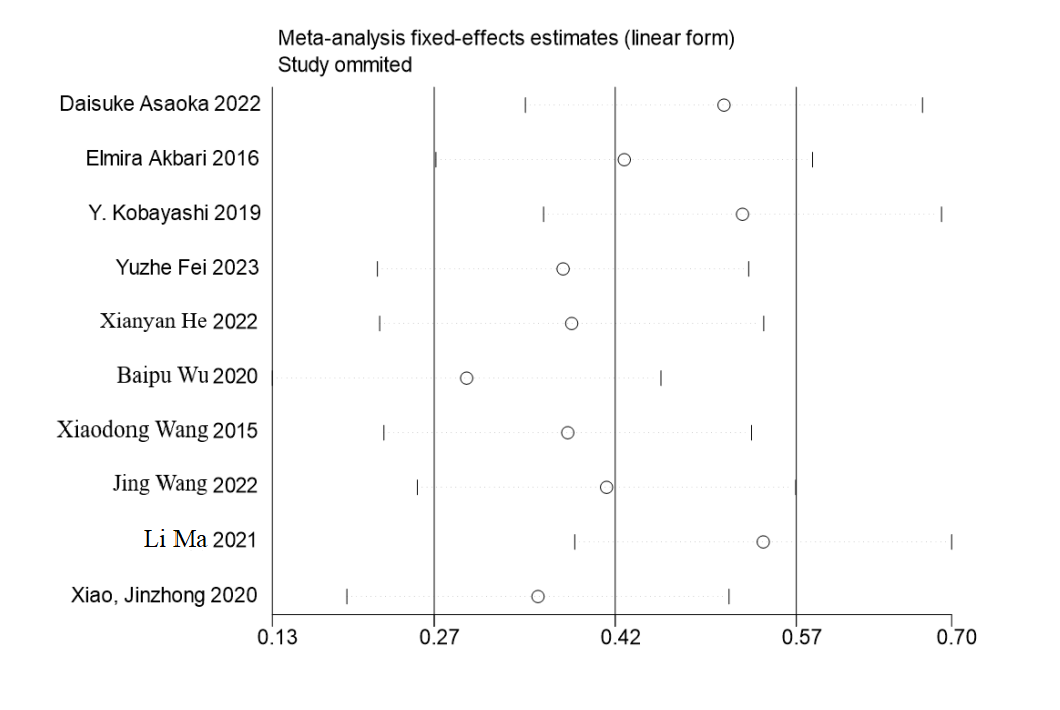
**
