# Supplementary material for: Efficacy of probiotics in patients with cognitive impairment: A systematic review and meta-analysis
Source: PLoS One. 2025 May 2;20(5):e0321567. doi: 10.1371/journal.pone.0321567 (PMC12047807; doi:10.1371/journal.pone.0321567)
Supplement: Attachment — Submitted filename: renamed_9781f.docx [file pone.0321567.s009.docx]

In this systematic review and meta-analysis, the impact of probiotics on patients with cognitive impairment was evaluated. Conclusion: Probiotics have been proven to enhance cognitive function, and a probiotic intervention program containing a single probiotic strain with a duration of<12 weeks has shown particularly strong effects in evaluating cognitive function through Mini Mental State Examination (MMSE). Some possible mechanisms have also been proposed in this regard. After careful evaluation, I found that most of the criteria for the review and meta-analysis of this manuscript have been taken into account. However, recheck the manuscript using the PRISMA checklist and revise or complete it to ensure that all standards have been met.

This paper is very interesting, but the following modifications still need to be made.

1.We noticed that the articles included in this review were from the establishment of the database until September 2024. But we noticed that the articles included by the researchers were mostly from after 2015. Based on your search strategy and search time, have you conducted sufficient searches on various databases, and were there any relevant articles before 2015? For what reason was it not included?

2.In the subgroup analysis, separate analyses were conducted on the scales used, intervention time, and types of probiotics. We noticed that the age range of the researchers included in this study was between 50-85 years old. As aging and aging are themselves high-risk factors for cognitive decline, no age-related subgroup analysis was conducted to determine the reasons for this.

3.The author order in Tables 3 and 4 should be based on alphabetical order or the year of publication of the article.

4.In the discussion, we found that sometimes cognitive impairment is used, but later it is also described in patients with Alzheimer's disease. Please clarify and distinguish the concepts of the two, pay attention to the theme of this article, and maintain consistency before and after.

5. In the discussion, if you can compare your findings with other intervention measures and ultimately determine the efficiency of your findings, it will be more convincing

6. This study found that the underlying theory needs a clearer explanation.

Some sentences are too long to understand and keep up with.
